# Chromatic vision and structural assessment in primary congenital glaucoma

**DOI:** 10.1038/s41598-024-60320-2

**Published:** 2024-04-25

**Authors:** Renata Tiemi Kato, Christiane Rolim-de-Moura, Norma Allemann

**Affiliations:** Department of Ophthalmology and Visual Sciences, Federal University of São Paulo, Escola Paulista de Medicina, Rua Botucatu, 806 – Secretaria Administrativa, São Paulo, SP CEP 04023-062 Brazil

**Keywords:** Ocular hypertension, Optic nerve diseases

## Abstract

Primary congenital glaucoma is a rare disease that occurs in early birth and can lead to low vision. Evaluating affected children is challenging and there is a lack of studies regarding color vision in pediatric glaucoma patients. This cross-sectional study included 21 eyes of 13 children with primary congenital glaucoma who were assessed using the Farnsworth D-15 test to evaluate color vision discrimination and by spectral domain optical coherence tomography to measure retinal fiber layer thickness. Age, visual acuity, cup-to-disc ratio and spherical equivalent data were also collected. Global and sectional circumpapillary and macular retinal fiber layer thicknesses were measured and compared based on color vision test performance. Four eyes (19%) failed the color vision test with diffuse dyschromatopsia patterns. Only age showed statistical significance in color vision test performance. Global and sectional circumpapillary and macular retinal fiber layer thicknesses were similar between the color test outcomes dyschromatopsia and normal. While the color vision test could play a role in assessing children with primary congenital glaucoma, further studies are needed to correlate it with damage to retinal fiber layer thickness.

## Introduction

Primary congenital glaucoma occurs at a rate of 1.5–3.4 per 100,000 births^1,2^. Clinical features such as epiphora, photophobia, corneal edema, Haab striae, increased corneal diameter, increased globe axial length and optic nerve cupping are typical and may be present at birth or early infancy^3,4^. Over 75% of congenital glaucoma eyes have intraocular pressure control with angle surgeries^5,6^, and 60% achieve visual acuity better than 20/60^2,7,8^. Correction of refractive errors, intraocular pressure control, monitoring of cup-to-disc ratio, axial length, perimetry and optical coherence tomography (OCT) are important for follow-up. Due to poor cooperation and photophobia, intraocular pressure measurements and refraction tests are often performed under general anesthesia^3^. Visual acuity and perimetry are the most common psychophysical tests used to evaluate glaucoma severity in children and adults^9^. However, perimetry has limitations in children due to lack of familiarity and comprehension of the exam, duration, maintaining correct position, and fatigue^10^. Other psychophysical tests such as color vision discrimination could be considered. Tsamparlakis et al.^11^ first described color vision defects as an anarchic pattern of dyschromatopsia in 10 children with successfully treated primary congenital glaucoma. Since then, there has been a lack of studies and color vision is not usually tested in children with glaucoma.

Pediatric glaucoma presents concentric damage in circumpapillary retinal fiber layer by OCT^12–15^. Although intraocular pressure control in primary congenital glaucoma eyes can reverse cupping, the damage is permanent^16^. Primary congenital glaucoma eyes also present thinning in macular retinal fiber layer thickness^17,18^. However, capturing OCT images in the eyes of children with glaucoma can be challenging due to low vision and potential lack of cooperation^18^.

This study aims to assess color vision using Farnsworth D-15 test and retinal fiber layer (RNFL) thickness using OCT in children with primary congenital glaucoma and to correlate color vision test performance to structural assessment by OCT.

## Patients and methods

In this cross-sectional study, we enrolled patients diagnosed with primary congenital glaucoma at the Pediatric Glaucoma Service of Federal University of São Paulo Hospital from 2018 to 2020. The study was approved by the Institutional Research Ethics Review Committee of Federal University of São Paulo Hospital (0756/2018) and was conducted in accordance with the Declaration of Helsinki. Written informed consent was obtained from all patients and their guardians. Inclusion criteria were primary congenital glaucoma diagnosis, age between five to twelve years old and visual acuity better than 0.1 (decimal) in at least one eye. Exclusion criteria included corneal opacification, congenital cataracts, other developmental or secondary glaucoma, optic neuritis, retinal diseases, previous ocular trauma, exposure to metal such as mercury^19^ or usage of medications that could interfere with color vision (metronidazole, ethambutol, interferon, digoxin)^20^ or cognitive impairment that could compromise the color discrimination test. Medical records of 303 pediatric glaucoma patients were retrieved from January 2017 to December 2017 and 13 children meeting the inclusion criteria were recruited. Pediatric glaucoma subjects with intraocular pressure controlled below 21 mmHg by means of angle surgeries, drainage implant surgery, cyclophotocoagulation or hypotensive eyedrops were included in the study. Non-controlled glaucoma cases were excluded, because high intraocular pressure could lead to corneal modifications (keratopathy) and bias the color vision performance.

Comprehensive ophthalmologic exam was performed by a single examiner (RTK), including visual acuity, color vision test, slit lamp biomicroscopy, intraocular pressure measurement with an Icare®TA01i (Tiolat Oy, Helsinki, Finland) tonometer, dilated eye fundus exam, refraction, cup-to-disc ratio evaluation and OCT exam (details of the OCT instrument used and scan types acquired are provided below).

### Color vision assessment

Farnsworth D-15 test (Quantitative color vision test; Precision Vision, USA) was used to evaluate color vision. Fifteen pseudoisochromatic plates, numbered on the backside, were presented at a reading distance of approximately 30 cm, with the best correction and monocularly, in an illuminated room using a 30-W fluorescent lamp. The child was required to sort the plates according to a progressive chromatic tonality. The result sequence characterized the color vision as normal or indicating diffuse, tritan, protan or deutan defects.

### Optical coherence tomography

Optic disc and macular scanning were performed using spectral domain OCT Spectralis (Heidelberg Engineering, 2015, USA). For circumpapillary retinal fiber layer (cpRNFL) thickness measurements, a circle of 3.6 mm diameter centered at the optic nerve head was projected on the retina and circular scans were performed. The Spectralis OCT software divided the circumpapillary retinal fiber layer thickness map into 7 sectors: average, superior-temporal, temporal, inferior-temporal, inferior-nasal, nasal and superior-nasal. For macular segmentation, “fast macular cube” protocol was used, an area of 5.8 mm × 5.8 mm centered at the fovea was analyzed using 25 B-scans separated by 242 microns. For macular thickness measurements, the layers were defined according to the Early Treatment of Diabetics Retinopathy Study (ETDRS) map: 1 mm ring centered in the macula as the fovea, 3 mm and 6 mm rings as inner and outer rings, respectively. Each of these rings was divided into four sectors defined as superior, nasal, inferior and temporal.

### Statistical analysis

Statistical analyses were performed using SPSS Statistics v.28.0.1.0 (IBM Corp., USA). Percentages were calculated for categorical variables, means and standard deviations (SDs) were calculated for parametric numerical data. Medians and ranges were determined for the nonparametric numerical data. Visual acuity measurements were converted to decimal format.

A web-based software^21^ was used to convert the Farnsworth D-15 test color sequence into a graph. The result was classified as normal, diffuse defect, possible protanopia, possible deuteranopia and possible tritanopia using the Moment of Inertia method^22^. Since only normal and diffuse defect responses were obtained in this study, the color test outcome variable was analyzed as a dichotomous variable. Measurements for circumpapillary retinal fiber layer and macular retinal fiber layer thicknesses are reported in micrometers (µm). The nonparametric Mann–Whitney U test for independent samples was employed to assess the correlation between the dichotomic categorical variable (color vision test outcome) and the numerical variables (circumpapillary retinal fiber layer and macular retinal fiber layer thicknesses measurements). Generalized linear model (GLM) using generalized estimation equation (GEE) was used to evaluate the subjects. The within-subject variable was laterality (right or left eye), the dependent variable was color vision test outcome, and the covariates were age, visual acuity, spherical equivalent and cup-to-disc ratio. The results were presented as odds ratio (OR) and 95% confidence interval. A *p* value < 0.05 was considered statistically significant.

## Results

**Figure 1:**
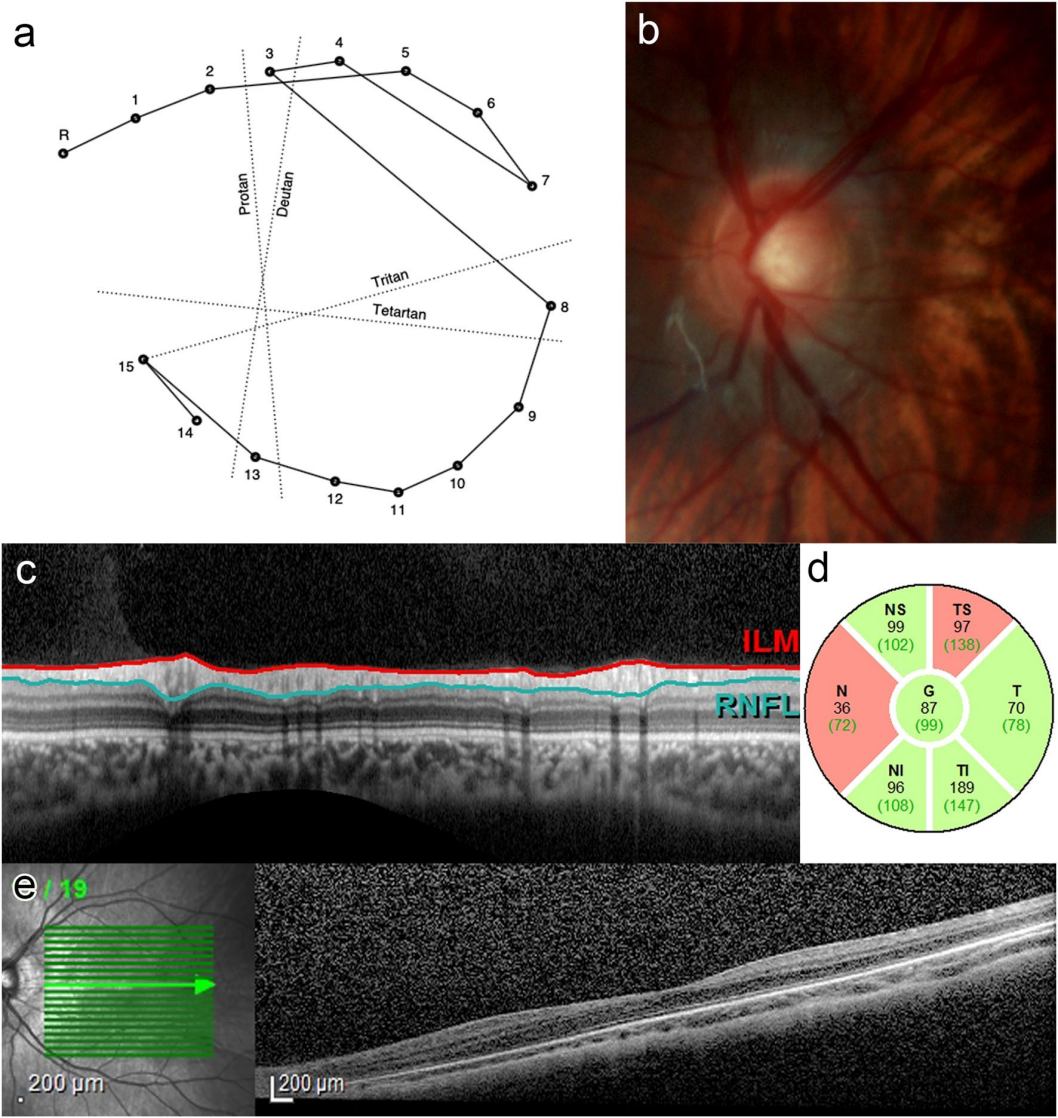
Exams in child with primary congenital glaucoma. (**a**) Diffuse defect in color vision by Farnsworth-D15 test. Number sequence represents pseudoisochromatic plaques sorted by the child. (**b**) Optic disc retinography. (**c**) Optic disc retinal fiber layer thickness by OCT. (**d**) Optic disc thicknesses measurements divided into 7 sectors: average (G), temporal-superior (TS), temporal (T), temporal-inferior (TI), nasal-inferior (NI), nasal (N), nasal-superior (NS). Numbers in black are the patient’s thickness and numbers in green parenthesis, the average for each sector. Green-filled sectors are considered within normal limits (G, T, TI, NI) and red-filled sectors are below average (TS, N). (**e**) Macular retinal fiber layer thickness by OCT.

Twenty-one eyes of 13 subjects were enrolled and all presented bilateral primary congenital glaucoma. Five eyes were excluded due to low vision (worse than 0.1). The mean age was 8.3 ± 2.0 years old, the mean cup-to-disc ratio was 0.6 ± 0.3, the median spherical equivalent was − 3.8 ± 5.3 D and the median decimal Snellen visual acuity was 0.54 ± 0.3. All subjects underwent the Farnsworth D15 test. Four eyes (19%) failed the color vision test and exhibited diffuse dyschromatopsia. Figure 1 shows exams in a child with primary congenital glaucoma and color vision test defect. Circumpapillary retinal fiber layer thickness maps were feasible in 14 eyes (66.7%) and macular retinal fiber layer thickness maps in 10 eyes (47.6%). Among the 7 eyes that failed in optic disc OCT image capturing compared to 14 eyes that successfully had images, mean age was 8.4 ± 1.6, and 8.3 ± 2.2 years old, mean visual acuity was 0.4 ± 0.3 and 0.6 ± 0.3, mean cup-to-disc ratio was 0.7 ± 0.3 and 0.6 ± 0.3 and median spherical equivalent was − 7.9 ± 6.6D e − 1.4 ± 2.4D, respectively. Considering the 11 eyes which failed in macular OCT image capturing compared to the 10 eyes that successfully had images, mean age was 8.3 ± 1.7 and 8.4 ± 2.4 years, mean visual acuity was 0.5 ± 0.4 and 0.6 ± 0.3, mean cup-to-disc ratio was 0.6 ± 0.3 and 0.6 ± 0.2, and median spherical equivalent was − 5.0 ± 6.5D and − 2.1 ± 2.7D respectively. Table 1 shows demographic and ophthalmologic characteristics, color vision test outcomes alongside optic disc and macular retinal fiber layer thicknesses measurements by OCT.

**Table 1:** Color vision test outcomes (diffuse defect and normal) alongside retinal fiber layer thickness values (microns) by OCT in children with primary congenital glaucoma, and corresponding eye characteristics: age (years), visual acuity (decimal), cup to disc ratio and spheric equivalent (diopters).

Patient		Eye characteristics				Color test outcome	Retinal fiber layer thickness by OCT in microns																
		Age	VA	CDR	SE		Optic disc							Macular									
		ys	dec		dp		Ave	Sup	Temp	InfT	InfN	Nas	SupN	OS	IS	OT	IT	OI	II	ON	IN	Fov	Vol
1	RE	9	0.25	0.9	− 17.0	Normal	–	–	–	–	–	–	–	–	–	–	–	–	–	–	–	–	–
	LE	9	0.20	0.8	− 13.0	Normal	–	–	–	–	–	–	–	–	–	–	–	–	–	–	–	–	–
2	RE	8	0.10	0.9	− 1.0	Normal	47	66	33	38	44	55	50	289	311	273	298	268	303	291	301	253	8.05
	LE	8	0.80	0.8	− 1.0	Normal	66	64	48	114	80	63	51	301	331	290	319	308	331	313	335	265	8.07
3	RE	8	0.67	0.6	− 3.5	Normal	55	73	48	28	52	71	52	291	335	275	310	274	321	317	347	259	8.40
	LE	8	0.50	0.6	− 0.5	Normal	94	114	60	121	108	86	114	305	344	289	331	301	345	336	346	262	8.87
4	RE	9	0.80	0.6	− 3.0	Normal	102	157	76	137	136	61	114	316	320	295	318	286	336	295	320	257	8.55
	LE	9	0.20	0.7	− 8.0	Defect	87	97	70	189	96	36	99	397	408	354	373	380	454	355	401	311	10.70
5	RE	5	0.10	0.8	0	Defect	59	69	60	44	64	60	52	287	274	262	248	–	256	287	258	219	6.23
	LE	5	1.00	0.6	0	Normal	78	115	73	105	46	63	88	312	333	292	317	304	324	324	325	245	8.77
6	LE	10	0.80	0.7	− 1.0	Normal	–	–	–	–	–	–	–	–	–	–	–	–	–	–	–	–	–
7	RE	9	1.00	0.2	0	Normal	99	143	62	135	111	83	112	–	–	–	–	–	–	–	–	–	–
	LE	9	1.00	0.2	0	Normal	98	131	66	138	116	75	119	–	–	–	–	–	–	–	–	–	–
8	LE	8	0.17	0.9	2.0	Normal	–	–	–	–	–	–	–	–	–	–	–	–	–	–	–	–	–
9	LE	9	0.67	0.4	0	Normal	79	71	62	124	119	65	66	–	–	–	–	–	–	–	–	–	–
10	RE	9	1.00	0.8	− 9.0	Normal	–	–	–	–	–	–	–	–	–	–	–	–	–	–	–	–	–
	LE	9	0.20	0.9	− 7.0	Normal	–	–	–	–	–	–	–	–	–	–	–	–	–	–	–	–	–
11	RE	12	0.80	0.2	0	Normal	96	122	65	146	114	82	93	298	336	286	325	288	333	319	331	255	8.60
	LE	12	0.67	0.2	0	Normal	98	112	57	144	127	94	98	304	333	284	327	286	336	320	337	266	8.63
12	RE	5	0.13	0.9	0	Defect	64	67	59	90	89	34	77	–	–	–	–	–	–	–	–	–	–
13	RE	5	0.40	0.9	− 10.0	Defect	–	–	–	–	–	–	–	–	–	–	–	–	–	–	–	–	–

*OCT* ocular coherence tomography, *RE* right eye, *LE* left eye; Age in years, *VA* visual acuity in decimal, *CDR* cup-to-disc ratio, *SE* spheric equivalent in diopters, *Ave* average, *Sup* superior, *Temp* temporal, *InfT* inferior-temporal, *InfN* inferior-nasal, *Nas* nasal, *SupN* superior-nasal, *OS* outer-superior, *IS* inner-superior, *OT* outer-temporal, *IT* inner-temporal, *OI* outer-inferior, *II* inner-inferior, *ON* outer-nasal, *IN* inner-nasal, *Fov* fovea, *Vol* volume.

The logistic regression using the GEE model revealed a correlation between the dependent variable color vision test outcome and the variable age (OR: 0.87, CI 0.79–0.95) in Table 2. There was no correlation between the color vision test outcome and the variables visual acuity (OR: 0.69, CI 0.42–1.15), spherical equivalent (OR: 0.99, CI 0.96–1.03) and cup-to-disc ratio (OR: 1.06, CI 0.58–1.95).

**Table 2:** Logistic regression analysis using GEE* for demographic and ophthalmological factors associated to color vision test outcomes (dependent variable).

	Odd ratio	95% CI	*p* Value*
Age, years	0.87	0.79–0.95	0.002
Spherical equivalent, dioptric	0.99	0.96–1.03	0.713
Visual acuity, decimal	0.69	0.42–1.15	0.155
Cup-to-disc ratio	1.06	0.58–1.95	0.839

*Generalized estimation equation (GEE).

Table 3 presents compares circumpapillary retinal fiber layer thicknesses according to the color vision test outcomes (diffuse defect and normal) and there was no difference between outcomes in circumpapillary retinal fiber layer thicknesses, except for the nasal sector (*p* = 0.01). Table 4 presents macular retinal fiber layer thicknesses (in microns) according to the color vision test outcomes (diffuse defect and normal) and demonstrates no difference between outcomes in macular retinal fiber layer thicknesses.

**Table 3:** Circumpapillary retinal fiber layer thickness (in microns) according to the color vision test outcomes (diffuse defect and normal).

cp RNFL thickness	Color vision test outcomes		*p* Value*
	*Diffuse defect*	*Normal*	
	*N* = *3*	*N* = *11*	
	*Mean in microns (SD)*	*Mean in microns (SD)*	
Global	*70.00 (14.93)*	*82.91 (19.41)*	0.29
Sup-temporal	*77.67 (16.77)*	*106.18 (32.73)*	0.23
Temporal	*63.00 (6.08)*	*59.09 (12.29)*	0.89
Inf-temporal	*107.67 (76.10)*	*111.82 (41.01)*	0.89
Inf-nasal	*83.00 (16.82)*	*95.73 (34.03)*	0.46
Nasal	*43.33 (14.47)*	*72.55 (12.36)*	0.01
Sup-nasal	*76.00 (23.52)*	*87.00 (27.56)*	0.66

*Mann–Whitney U.

Italics correspond to the thicknesses.

**Table 4:** Macular retinal fiber layer thickness (in microns) according to the color vision test outcomes (diffuse defect and normal).

m RNFL thickness	Color vision test outcomes		*p* Value*
	*Diffuse defect*	*Normal*	
	*N* = *2*	*N* = *8*	
	*Mean in microns (SD)*	*Mean in microns (SD)*	
Outer-superior	*342.00 (9.31)*	*302.00 (77.78)*	1.00
Inner-superior	*341.00 (94.75)*	*330.38 (10.25)*	1.00
Outer-temporal	*308.00 (65.05)*	*285.50 (7.87)*	1.00
Inner-temporal	*310.50 (88.39)*	*318.13 (10.45)*	1.00
Outer-inferior	*380.00 (–)*	*289.38 (14.21)*	0.22
Inner-inferior	*355.00 (140.01)*	*328.63 (12.75)*	1.00
Outer-nasal	*321.00 (48.08)*	*314.38 (14.85)*	1.00
Inner-nasal	*329.50 (101.16)*	*330.25 (15.05)*	1.00
Foveal	*265.00 (65.05)*	*257.75 (6.90)*	1.00
Volume	*8.47 (3.16)*	*8.49 (0.30)*	1.00

*Mann–Whitney U.

Italics correspond to the thicknesses.

## Discussion

Color vision defects can impact daily life, affecting even routine tasks such as selecting ripe fruit or fresh meat, discerning health and emotional states from skin color, and identifying traffic lights colors. Additionally, career choices may be influenced by color vision, underscoring the importance of early dyschromatopsia diagnosis^23,24^. While there is a variety of color vision tests, none are entirely suited for children^25^. The Ishihara test is the most widely used test worldwide, but it only detects color vision defects and does not classify them, besides it has low specificity in children. Panel tests like the Farnsworth D15 and Farnsworth Munsell 100-hue offer potential for diagnosing and classifying color vision deficiencies. The Farnsworth D15 test was used in this study because it is a less time-consuming color vision test, typically taking around 3 min per eye, compared to the Farnsworth-Munsell 100-Hue test, which takes 20 min per eye and is not recommended for children^26^. Children are not routinely tested for color vision, and there is a shortage of information about color vision, even in healthy children. Shrestha et al.^27^ has demonstrated that between the ages of 8–15 years old, both the Ishihara test and D15 test exhibits similar capacity in detecting color vision defects. Using D15 test, Pinckers^28^ described a 9% diffuse defect in normal color vision children aged 0–9 years old and only minor defects detectable in those above 10 years old.

While previous studies have focused on congenital dyschromatopsia^29,30^, little is known about acquired dyschromatopsia due to conditions like glaucoma. The prevalence of color vision defects in children with primary congenital glaucoma was found to be 19% in this study, with all four cases occurring in male subjects and characterized as diffuse defect type. There was no child with color deficiency in both eyes, so we assume the color deficiency reported is related to acquired condition. Younger children performed worse than older ones, consistent with the expected higher error score on the Farnsworth D15 test for young children^28^. Other studies have also shown that color vision tends to improve with age from childhood to adolescence in panel tests^31–33^. This improvement could be related not only to ocular maturation but also to the maturation of attention and cognitive skills. Our sample narrowed mostly due to poor vision. Lopes et al.^34^ reported that 80% of all childhood glaucoma patients present at our service in an advanced stage of the disease, with a cloudy cornea and cup-to-disc ratio over 0.8. Even so, visual acuity, spherical equivalent and cup-to-disc ratio did not influence the color vision discrimination test.

Unlike primary open-angle glaucoma-associated dyschromatopsia, which mostly presents as tritanopia^35^, congenital glaucoma-associated diffuse dyschromatopsia could be related to the timing of increased intraocular pressure. Recent studies indicate that neonates have color perception, although it is reduced and still developing^36^. Histological studies show that before birth, the foveola could be more than 1000 µm thick, but it reduces to 650–700 µm at 4 years old with cone migration to the central region^37^. An intraocular pressure peak at birth or in the first months of life could interfere with migration, quality and connection of cones and consequently with the ability to distinguish colors. In future studies, it would be interesting to document the age at diagnosis of the disease, age of treatment, and correlation with color tests to assess whether the moment of treatment is related to damage in the migration of the cones and color vision impairment.

Although the present study showed a slightly higher prevalence of color vision defects in children with congenital glaucoma, glaucoma is not the most common disease when evaluating a child with color vision defect. Tirpack et al.^38^ evaluated pediatric glaucoma suspects referred for neuro-ophthalmological investigation. The most common reasons for referral were optic disc pallor, color vision defect and retinal fiber layer thinning by OCT. The investigation concluded that 51.7% of patients who had color vision defect and/or retinal fiber layer thinning < 70 µm by OCT had an ultimate neurologic diagnosis. Therefore, in children without typical glaucoma clinical findings, with color vision defects and retinal fiber layer thinning by OCT, it is mandatory to investigate neuro-ophthalmological diseases.

Previous studies measured retinal fiber layer thickness by OCT in other color vision pathologies. Largueche et al.^39^ evaluated 6 achromatopsia patients, and OCT exam showed a loss of inner and outer segments, as well as the appearance of partial-thickness holes in the outer macular retina. Oszoy et al.^40^ evaluated 22 individuals and Gupta et al.^41^ evaluated 7 patients with congenital dyschromatopsia, comparing retinal fiber layer thickness of congenital dyschromatopsia and normal subjects. They showed no difference in circumpapillary and macular retinal fiber layer thicknesses between the groups. Prior work also measured retinal fiber layer thickness in other optic nerve pathology. Zahavi et al.^42^ evaluated 23 children with optic pathway glioma, 10 with and 13 without neurofibromatosis, measured the average retinal fiber layer thickness, tested color vision using Ishihara test and concluded that thinning in retinal fiber layer thickness was associated with a decrease in color vision. In the present study, macular or circumpapillary retinal fiber layer thickness could not distinguish glaucoma-acquired dyschromatopsia from normal color vision, except for the nasal circumpapillary sector. However, the correlation between groups for the nasal sector of circumpapillary retinal fiber layer thickness could be biased by the small sample size.

This study has limitations, it is a small observational study with no control group and missing OCT data. Three out of four children failed in color vision test at age 5, and they may not have been able to collaborate due to young age. It is possible they could perform better as they age beyond 10 years old. Ocular coherence tomography assessment was not possible for all children in the study, likely due to a lack of cooperation. When comparing the groups: eyes of children whose OCT exam was successfully and unsuccessfully performed, mean age, visual acuity, spherical equivalent and cup-to-disc ratio were similar. In other words, it was not due to low visual acuity, high ametropia, young age or advanced disease that hampered the OCT exam. It is possible that the lengthy assessment conducted on a single day may have exhausted the children's attention. In a new study, recruiting a larger sample size, including a control group, conducting the OCT exam on a different day as an alternative to avoid fatigue and following up by repeating the color vision test could help avoid potential biased conclusions.

To date, this is the first study to attempt to correlate color vision performance with retinal fiber layer thickness using OCT in children’s eyes with primary congenital glaucoma. The assessment of children with glaucoma is challenging, as many tests require their collaboration and sometimes even necessitate general anesthesia for a reliable examination. The Farnsworth D15 color vision test is a noninvasive and low-cost psychophysical modality. In this study, the Farnsworth D15 test could be easily performed in children with congenital glaucoma over 5 years old with visual acuity better than 0.1, even in cases of high ametropia and advanced disease. Younger children tended to perform worse on the test. There was no correlation between color vision test outcome and circumpapillary and macula retinal fiber layer thicknesses measured by OCT. Although the color vision test could play a role in assessment of children with primary congenital glaucoma, further studies are needed to correlate it with damage to the retinal fiber layer by OCT.

## Data Availability

The datasets generated during and/or analyzed during the currently study are available from the corresponding author on reasonable request.
